# Intravenous Administration of Human Muse Cells Ameliorates Deficits in a Rat Model of Subacute Spinal Cord Injury

**DOI:** 10.3390/ijms241914603

**Published:** 2023-09-27

**Authors:** Yoshiharu Takahashi, Takumi Kajitani, Toshiki Endo, Atsushi Nakayashiki, Tomoo Inoue, Kuniyasu Niizuma, Teiji Tominaga

**Affiliations:** 1 Department of Neurosurgery, Graduate School of Medicine, Tohoku University, Sendai 980-8572, Japan; ytakahashi0913@gmail.com (Y.T.); jopmagsvmnt@gmail.com (A.N.);; 2 Department of Neurosurgery, Tohoku Medical and Pharmaceutical University, Sendai 981-8558, Japan; 3 Department of Neurosurgery, Saitama Red Cross Hospital, Saitama 330-8553, Japan; tomoo49@gmail.com; 4 Department of Neurosurgical Engineering and Translational Neuroscience, Graduate School of Medicine, Tohoku University, Sendai 980-8576, Japan; 5 Department of Neurosurgical Engineering and Translational Neuroscience, Graduate School of Biomedical Engineering, Tohoku University, Sendai 980-8572, Japan

**Keywords:** muse cell, intravenous administration, rat, spinal cord injury

## Abstract

Multilineage-differentiating stress-enduring (Muse) cells are newly established pluripotent stem cells. The aim of the present study was to examine the potential of the systemic administration of Muse cells as an effective treatment for subacute SCI. We intravenously administered the clinical product “CL2020” containing Muse cells to a rat model two weeks after mid-thoracic spinal cord contusion. Eight experimental animals received CL2020, and twelve received the vehicle. Behavioral analyses were conducted over 20 weeks. Histological evaluations were performed. After 20 weeks of observation, diphtheria toxin was administered to three CL2020-treated animals to selectively ablate human cell functions. Hindlimb motor functions significantly improved from 6 to 20 weeks after the administration of CL2020. The cystic cavity was smaller in the CL2020 group. Furthermore, larger numbers of descending 5-HT fibers were preserved in the distal spinal cord. Muse cells in CL2020 were considered to have differentiated into neuronal and neural cells in the injured spinal cord. Neuronal and neural cells were identified in the gray and white matter, respectively. Importantly, these effects were reversed by the selective ablation of human cells by diphtheria toxin. Intravenously administered Muse cells facilitated the therapeutic potential of CL2020 for severe subacute spinal cord injury.

## 1. Introduction

Spinal cord injury (SCI) induces local neural cell death and the disruption of axonal pathways, potentially causing devastating neurological damage to patients. However, neurological recovery is considered to be limited after SCI because the central nervous system (CNS) environment does not generally permit regeneration [1,2]. To compensate for local spinal cord damage, cell transplantation has emerged as a potential therapeutic approach [3]. Among many cell sources for regenerative medicine, the unique characteristics of multilineage-differentiating stress-enduring (Muse) cells, which are endogenous pluripotent stem cells, have attracted attention [4]. Muse cells exist in mesenchymal tissues, including bone marrow and adipose and dermal tissues. Since Muse cells express the pluripotent surface marker stage-specific embryonic antigen-3, they may be harvested and cultured as a source for regenerative medicine [5]. Besides SSEA-3, Muse cells express pluripotency markers including Oct3/4 and Sox2 along with other markers known to be expressed by mesenchymal stem cells including CD105, CD90, and CD29 [6], since Muse cells are a specific subpopulation of human mesenchymal stem cells. A single Muse cell can generate cells representative of the three germ layers [5]. Muse cells exhibit low telomerase activity and are rarely tumorigenic [7]. In an experimental acute myocardial infarction model, the infarct size and functional recovery were significantly better in the Muse cell treatment group when compared to those of the mesenchymal stem cell group [8]. In a stroke model, Muse cell transplantation improved behavioral scores more significantly than mesenchymal stem cells [9].

We recently performed experimental SCI studies using CL2020 (Life Science Institute, Inc., Tokyo, Japan), which is a Muse-cell-based product produced from human mesenchymal stem cells. When CL2020 was intravenously administered one day after SCI, spinally injured animals showed better functional recovery from their neurological deficits [10]. We demonstrated that Muse cells in CL2020 recognized and migrated to the injured site. Furthermore, Muse cells differentiated into neuronal cells and exerted neuroprotective and regenerative effects in acute SCI.

In the present study, we administered CL2020 2 weeks after SCI, which we called the subacute SCI model. In a previous experiment [10], we examined the therapeutic potentials of Muse cells when they were administered acutely after SCI. However, having considered that the immediate post-injury phase has a hostile environment for transplanted cells, subacute cell transplantation therapy may be more applicable to human SCI patients [11]. In this current experiment, we intended to examine whether subacute Muse cell administration would still have positive effects on SCI. Behavioral and histological evaluations were performed to confirm the therapeutic effects of CL2020 against subacute SCI.

## 2. Results

### 2.1. Intravenous CL2020 Improves Hindlimb Locomotor Functions after Subacute SCI

**Figure 1:**
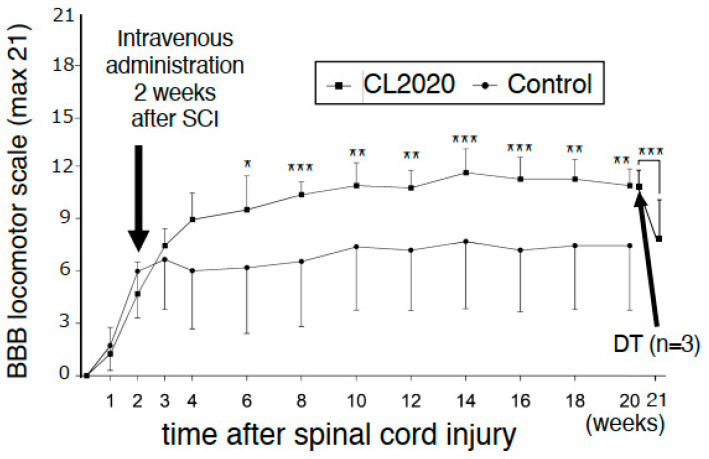
Basso, Beattie, and Bresnahan (BBB) locomotor scores. The open-field locomotor hindlimb function of all rats was tested on the day after injury and weekly thereafter for 20 weeks. CL2020 was intravenously administered two weeks after the injury. Results are shown separately for the CL2020 (line with square markers, *n* = 8) and Control groups (line with circle markers, *n* = 12). Six weeks after the injury, BBB scores were significantly higher in the CL2020 group than in the Control group. In three animals from the CL2020 group, diphtheria toxin (DT) was administered to revert engrafted cell functions. In the CL2020 group, BBB scores significantly decreased 5 days after the administration of DT. *: *p* < 0.05, **: *p* < 0.01, ***: *p* < 0.001.

Rats received CL2020 two weeks after SCI. BBB locomotor scores for the CL2020 and Control groups were determined each week for 4 weeks after injury and every other week thereafter for 20 weeks. Improvements in hind limb motor function were significantly greater in the CL2020 group than in the Control group after 6 weeks (*p* < 0.05) and over 8–20 weeks (*p* < 0.01 or 0.001, repeated ANOVA measures followed by Bonferroni post hoc test, Figure 1). Twenty weeks after the injury, the average BBB scores were 10.9 ± 0.96 and 7.4 ± 3.1 in the CL2020 and Control groups, respectively (*p* < 0.01, Figure 1); the treated animals could support weight on their hindlimbs and take plantar steps. There were also occasional instances of coordination between the forelimbs and hindlimbs, signaling progression in the recovery process.

### 2.2. CL2020 Prevented Spinal Cord Damage and Contributed to Structural Preservation

**Figure 2:**
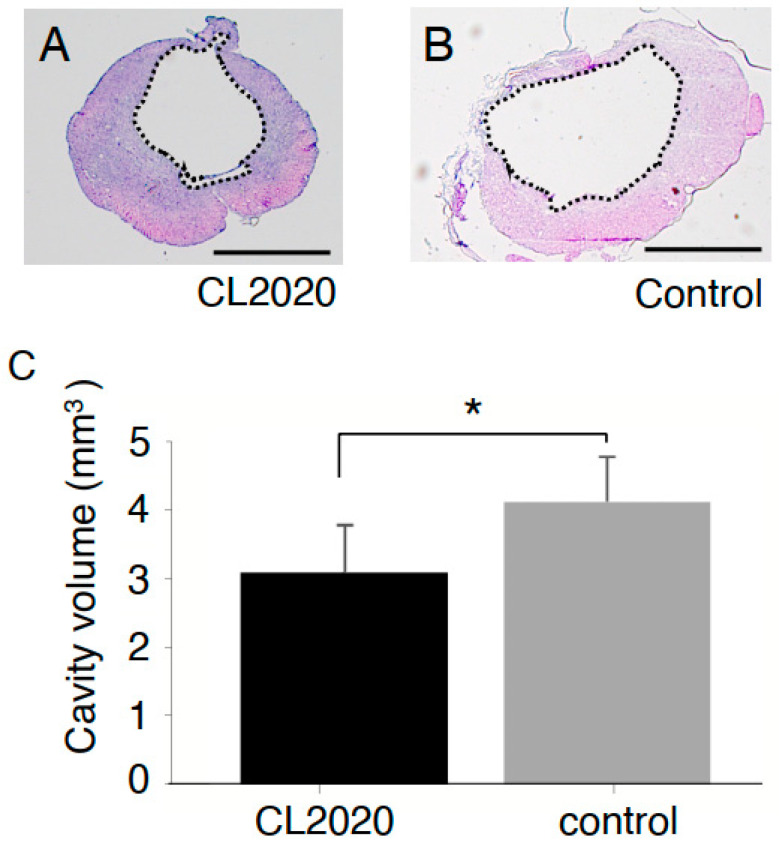
Measurements of the cystic cavity. Representative spinal cord axial sections in the lesion center of the CL2020 (**A**) and Control groups (**B**) in HE staining. Dotted lines indicate cavity areas in sections. The volumes of the cystic cavity were calculated as described in the Materials and Methods section. Scale bars = 1000 µm. (**C**) A graph showing cystic cavity volumes in the CL2020 (black) and control (gray) groups. Cavities were significantly smaller in the CL2020 group (*: *p* < 0.05).

The volumes of the cystic cavity in rats treated with CL2020 were significantly smaller than those in the Control group (*p* < 0.05, Figure 2). The average volumes of the cavity were 3.1 ± 0.69 and 4.1 ± 0.64 mm^3^ in the CL2020 and Control groups, respectively.

**Figure 3:**
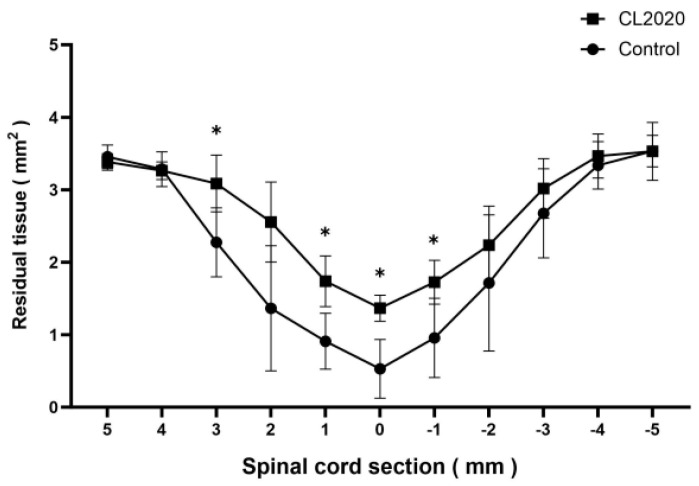
Areas of spared spinal cord tissue in relation to the lesion epicenter. Results are shown separately for the CL2020 (line with circle markers, *n* = 5) and Control groups (line with circle markers, *n* = 5). Twenty weeks after the injury, larger areas of the spinal cord were preserved in the CL2020 group than in the control group. *: *p* < 0.05.

**Figure 4:**
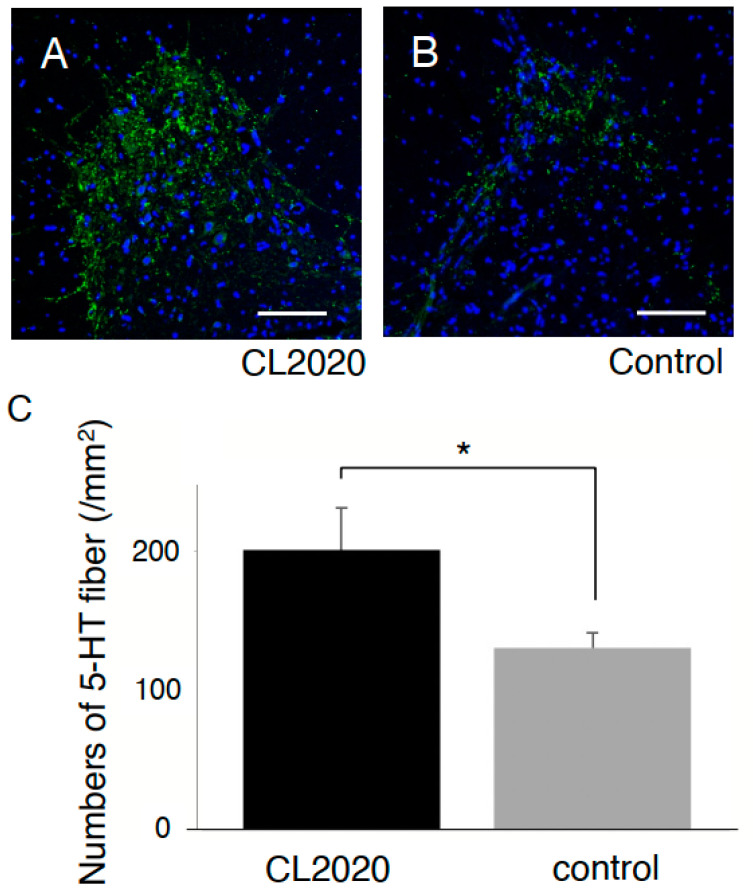
5-HT immunostaining. Representative figures of axial spinal cord sections 3 mm distal to the injury in the CL2020 (**A**) and Control groups (**B**) in the ventral horn of the spinal cord. 5-HT-positive fibers are stained green. Nuclei were counterstained with DAPI (blue). Scale bars = 100 µm. (**C**) A graph showing that the number of preserved 5-HT-positive fibers was larger in the CL2020 group (black) than in the Control group (gray) (*: *p* < 0.05). The figure is available in color online only.

The areas of the spared spinal cord tissue were significantly larger than those of the Control group 20 weeks after the injury (Figure 3). It was noted that larger areas of the spinal cord were preserved in the CL2020 group than in the Control group 5, 4, 3, and 1 mm rostral and 1 mm caudal to the lesion epicenter. Furthermore, larger numbers of 5-HT-positive axons were preserved in the ventral horn of the caudal spinal cord in the CL2020 group (*p* < 0.05, Figure 4). These results suggest that Muse cells in CL2020 exerted neuroprotection in the injured spinal cord.

### 2.3. CL2020 Engrafts and Differentiates into Neuronal Cells in the Injured Spinal Cord

Twenty weeks after the administration of CL2020, human cells in the injured spinal cord were identified via positive immunostaining using hMit in five CL2020-treated animals. No animals in the Control group showed cells positive for hMit. In CL2020-treated animals, positive cells were located in the lesion center and rostral and caudal perilesional areas in the spinal cord. In triple staining, hMit-positive cells expressing MAP-2, GFAP, or GST-pi were identified in the lesion center and rostral and caudal spinal cord. The percentages of these cell types in different locations of the spinal cord are listed in Table 1 (*n* = 5 in the CL2020 group). No significant differences were observed in the percentages of these three cell types. 

**Figure 5:**
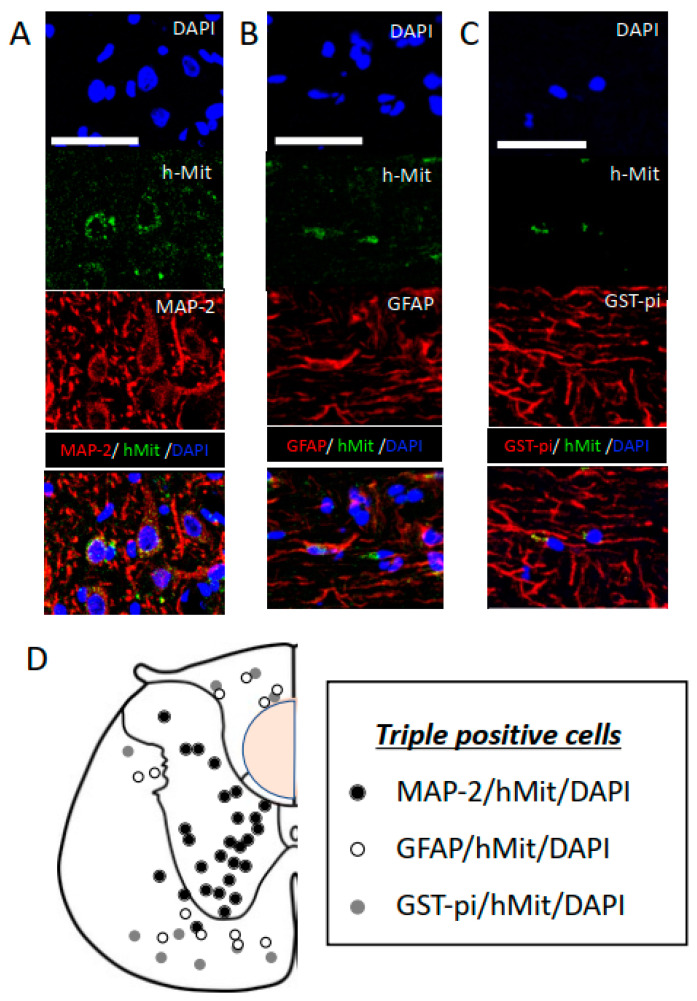
Differentiation of intravenously administered CL2020 in the injured spinal cord. (**A**–**C**) Higher-magnification histological images of engrafted cells 20 weeks after the spinal cord injury. Human cells were identified as hMit-positive cells (green). Nuclei were counterstained with DAPI (blue). In panel A, cells positive for MAP-2 (red) indicate neuronal differentiation. Cells positive for GFAP (red) and GST-pi (red) were also identified, indicating the astrocytic and oligocytic differentiation of hMit-positive cells in panels B and C, respectively. Bars = 50 μm. (**D**) The locations of MAP-2/hMit/DAPI- (black circles), GFAP/hMit/DAPI- (white circles), and GST-pi/hMit/DAPI- (gray circles) triple-positive cells are indicated in original illustrations (drawn by T.K.) of the axial sections of the unilateral spinal cord. MAP-2/hMit/DAPI-positive cells were predominantly located in the gray matter, and GFAP/hMit/DAPI- and GST-pi/hMit/DAPI-positive cells in the white matter. The orange circle indicates where the spinal cord was injured.

However, MAP-2/hMit/DAPI-positive cells were predominantly located in the gray matter and GFAP/hMit/DAPI- and GST-pi/hMit/DAPI-positive cells in the white matter (Figure 5). These results suggest that intravenously administered CL2020 homed into the injured spinal cord and predominantly differentiated into neuronal-marker-positive cells.

**Figure 6:**
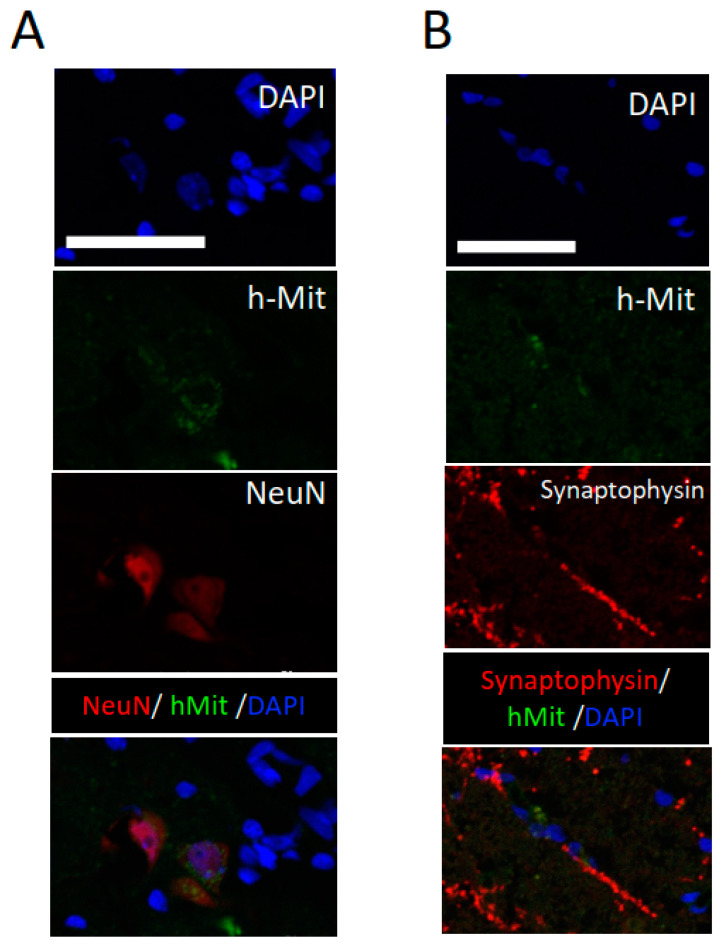
Higher-magnification histological images of engrafted cells 20 weeks after the spinal cord injury. (**A**) Differentiation of intravenously administered CL2020 to NeuN-positive cells in the injured spinal cord. Photomicrographs showing triple-positive cells with DAPI (blue), hMit (green), and NeuN (red). (**B**) Synaptophysin (red) was expressed around hMit-positive engrafted cells (green). Photomicrographs of a merged image are presented in the bottom panel. Scale bars = 50 μm.

Additionally, in the gray matter close to the lesion center, positive NeuN staining was recognized in hMit-positive cells. In the same area, synaptophysin positivity was noted in close relation to the hMit positive cells. These data support the neuronal differentiation of the engrafted cells (Figure 6).

### 2.4. Deterioration of Hindlimb Functions after the Administration of Diphtheria Toxin

A loss-of-function study was performed using diphtheria toxin to selectively cancel the functional improvements provided by CL2020 in spinally injured animals. Five days after the administration of diphtheria toxin, the BBB scores in the CL2020 group (*n* = 3) significantly decreased (*p* < 0.001, Figure 1). No significant differences were observed in the BBB scores in the Control group (*n* = 3) even after the administration of diphtheria toxin. Specifically, 5 days after the administration of diphtheria toxin, the BBB score dropped to 71.5 ± 12.8% (*p* < 0.001) in the CL2020 group, while it was 92.7 ± 4.2% in the Control group. These results suggest that CL2020 administration led to an improvement in the BBB score in the treatment group.

## 3. Discussion

The present study demonstrated that the administration of CL2020 significantly ameliorated neurological deficits in spinally injured experimental animals. When CL2020 was intravenously administered 2 weeks after SCI, Muse cells were considered to migrate to the injured spinal cord and differentiate into neuronal and neural lineage cells. Engrafted human Muse cells facilitated improvements in hindlimb motor functions over 20 weeks. When diphtheria toxin selectively ablated human Muse cell functions, the BBB scores of the spinally injured animals in the CL2020 group significantly deteriorated. The direct contribution of Muse cells to the functional recovery of hindlimb functions after SCI is supported by these results as well as histological evidence of a smaller cystic cavity and larger numbers of 5-HT fibers preserved distal to the injury.

### 3.1. Muse Cells Migrate to the Injured Spinal Cord

In this experimental model, hMit-positive cells were detected in the injured spinal cord 20 weeks after the intravenous administration of CL2020. This result suggests that Muse cells recognized the spinal cord as a damaged site and migrated there. As observed in many other organs, including the heart, liver, kidney, and brain [8,9,10,12,13], the homing phenomenon was confirmed in the spinal cord. In the current experimental protocol, we newly demonstrated Muse cell engraftment in the subacute SCI model after the intravenous administration of CL2020. Importantly, Muse cells survived in the injured spinal cord for as long as 20 weeks.

Using the same SCI model, we recently reported the migration of Muse cells to the damaged spinal cord when CL2020 was administered 1 day after the injury [10]. Collectively, these findings and the present results suggest that the intravenous administration of CL2020 is a reasonable approach for the delivery of Muse cells in different stages following SCI, including acute and subacute SCI. The long-term survival of intravenously administered Muse cells in the injured spinal cord reinforces the therapeutic potential of the administration of CL2020.

### 3.2. Mechanisms Underlying Muse Cell Migration and Homing

The mechanisms underlying the migration and homing of Muse cells following their intravenous administration are related to sphingosine-1-phosphate (S1P) [8]. S1P is a potent chemoattractant for neural stem/progenitor cells [8]. When cell membranes are injured, S1P is generated and functions as a signal of acute inflammation. In SCI, S1P concentrations reached the maximum levels 7 days after the injury [14]. Since Muse cells express S1P receptor 2 [15], they sense the trigger and migrate to the site of injury. These findings indicate that the S1P-S1P receptor 2 system is efficient in subacute treatment protocols. Hori et al. evaluated the systemic distribution of human Muse cells after intravenous injection in a hindlimb ischemia animal model [16]. They utilized human genomic DNA to detect the engrafted Muse cells, which led to the identification of the Muse cells in the injured limb, the lung, and the spleen, but not in the uninjured limb.

In a rabbit model of acute myocardial infarction, 14.5% of the administered Muse cells were shown to home to the injured heart and lung 3 days after intravenous injection [8]. However, after 2 weeks, the numbers of homed Muse cells in the heart were 10 times higher than those in the lung.

In considering the application of Muse cell transplantation therapy to the human condition, the immunotolerance of Muse cells is an important issue. Recent publications indicated that Muse cells have anti-immune mechanisms through the high expression of human leukocyte antigen G (HLA-G) and the immune modulatory enzyme indoleamine 2,3-dioxygenase (IDO) [8,17]. HLA-G is a potent immunosuppressing factor expressed by trophoblasts in the placenta that effectively demonstrates tolerogenic properties through interactions with inhibitory receptors on dendritic cells, natural killer cells, and T cells [18]. IDO also results in the inhibition of T cell proliferation through tryptophan degradation [19]. Through these mechanisms, Muse cells can achieve immune tolerance and represent a source for allogenic engraftments.

### 3.3. Muse Cells Differentiate into Neuronal Lineage Cells

Muse cells express pluripotency markers, such as Sox2, Oct3/4, and Nanog [4]. They spontaneously differentiate into tissue-compatible cells after homing to replenish new functional cells and repair damaged tissues [8,9,10]. In our subacute SCI model, Muse cells differentiated into various cells, including MAP-2-, NeuN-, GFAP-, and GST-pi-positive cells. As shown in Figure 5D, MAP-2/hMit-positive neuronal cells were predominantly located in the gray matter and GFAP/hMit- and GST-pi/hMit-positive cells in the white matter. Our results suggest that the differentiation of Muse cells is dependent on migration sites even within the spinal cord. When Muse cells were intravenously administered to an acute myocardial infarction model, they spontaneously differentiated into cardiac-marker-positive cells, including troponin-I and sarcomeric α-actinin [8]. Human Muse cells showed neuronal differentiation in the brain [9]. When Muse cells were transplanted into a rat stroke model, 65 and 30% of surviving engrafted cells were positive for NeuN and MAP-2, respectively. In the current protocol, more than 50% of surviving engrafted cells were positive for MAP-2 in the subacute SCI experiment. As recently reported, we confirmed the predominant neuronal differentiation of Muse cells in the injured spinal cord in an acute SCI experiment [10]. By comparing these two studies, the percentage of neuronal differentiation from CL2020 did not significantly differ based on the timing of administration. Since we detected larger numbers of surviving cells in the subacute SCI model, the subacute administration appeared to be more beneficial for ensuring higher survival rates of engrafted cells in the injured spinal cord (d). The present results are consistent with the subacute administration of neural stem cells supporting higher rates of graft survival and accommodation [11]. Immediately after SCI, a series of pathological and reactive alterations occur in the damaged spinal cord. Ischemia and necrosis make the injured spinal cord a hostile environment for transplanted cells to survive [20]. Prior to the application of the product CL2020 to future clinical trials on SCI, we consider it important to obtain evidence of its effects in an experimental subacute administration model.

### 3.4. Muse Cells Contribute to Functional Recovery

In the present study, the systemic administration of CL2020 induced significant functional improvements. We intended to selectively ablate transplanted human Muse cells using diphtheria toxin, as previously reported [10,21,22]. Theoretically, this method confirms whether Muse cells in CL2020 directly contribute to functional recovery. The BBB scores in the CL2020 group significantly decreased after the administration of diphtheria toxin to the subacute SCI model. This result indicated that Muse cells from CL2020 were directly associated with functional restoration after severe subacute SCI. SCI studies previously demonstrated that grafted neuronal cells make synaptic connections with endogenous neurons and reconstruct neuronal circuits in the spinal cord [3,20]. When a stroke model was intravenously administered Muse cells, the anterograde labeling of cortical neurons confirmed the reconstruction of the corticospinal tract [9]. Furthermore, the neurons originating from Muse cells were positively stained with a glutamatergic neuronal marker in the anterior horn of the spinal cord [9]. The results from an in vitro study also supported the notion of glutamatergic neuronal differentiation from Muse cells [23]. Oligodendrocytes are also expected to promote recovery through the remyelination of axons [24]. Although the underlying mechanisms have yet to be elucidated in detail, the present results support the potential of the product CL2020 as a reliable source for regenerative medicine in subacute SCI patients.

### 3.5. Limitations

The present results suggest the migration and homing of Muse cells to the injured spinal cord following their intravenous administration. We utilized a human mitochondria antibody to identify engrafted human cells in CL2020. While the antibody is expected to be specific to human mitochondria based on the previous literature [9,10,21], attention should be paid to its cross-reactivity with different species including rats, mice, and pigs [25]. Further, the mechanisms and the timing by which these cells reach the injured spinal cord and differentiate into neuronal and neural cells remain unclear. The transfer of differentiation-directing factors via the phagocytosis of apoptotic differentiated cells was proposed as a novel mechanism for Muse cell differentiation into target cell lineages [26]. We continue our research to demonstrate the fate of transplanted Muse cells, especially after diphtheria toxin ablation. This method can provide important explanations regarding the mechanisms of behavioral improvement after CL2020 treatment.

The time course of the differentiations in the injured rat spinal cord remains to be elucidated. In an in vitro study, Chen et al. demonstrated that neuronal differentiation of Muse cells was evident after 28 days of culture [23]. In a rat stroke model, differentiation of Muse cells was initiated shortly after administration. On day 3, the homed human Muse cells expressed early neuronal markers including Marsh1 and NeuroD. After 7 days, they expressed MAP-2, DCX, and NeuN [27]. Although we only observed Muse cell differentiation 20 weeks after the intravenous administration, it is important to know which fraction was present shortly after injection and in the following weeks when we consider the potential use of Muse cells for SCI therapy.

In this paper, we observed smaller cystic cavities in the injury center and larger numbers of preserved 5HT fibers in the CL2020-treated animals. What we report is limited to indirect evidence explaining behavioral improvements. For instance, identification of 5-HT-positive fibers in the spinal cord is used to support the hypothesis that Muse cells have neuroprotective effects. However, acute and subacute administration of CL2020 expressed the same degrees of neuroprotective effects after SCI [10]. We were not able to evaluate 5-HT immunolabeling on longitudinal sections and the capacity for axonal transport across the lesioned parenchyma. The mechanisms explaining the results remain to be established, which would eventually resolve an unanswered question regarding the best timing for Muse cell administration in SCI. Further studies are needed to establish how this new treatment should be applied to SCI.

## 4. Materials and Methods

### 4.1. Experimental Animals

The Animal Studies Ethics Committee of Tohoku University Graduate School of Medicine approved all animal experiments related to the present study. Efforts were made to minimize the numbers of animals used and decrease animal suffering in the experiments. Specifically, our researchers were well trained to minimize variability and ensure the quality of the data, which helped to minimize the number of animals. Further, we checked the animals frequently to minimize their discomfort by detecting early signs of stress or pain. Twenty-two adult female Sprague Dawley rats (body weight, 200–230 g) were used (Japan SLC, Inc., Shizuoka, Japan). Two or three rats were housed per cage and kept at a temperature of 24 °C with water and food ad libitum throughout the experiments.

### 4.2. SCI

The rats were anesthetized with 2% isoflurane in 30% oxygen and 70% nitrous oxide. The T9 spinous process was palpated on the top of the back while the rats were positioned comfortably. During surgery, the rectal temperature was maintained at 37.0 ± 0.5 °C using a feedback-regulated heating pad (BWT-100, Bio Research Center, Nagoya, Japan). Skin above the T9 spinous process was shaved and cleaned in an antiseptic manner. T9 laminectomy was performed after a midline dorsal skin incision, which widely exposed the dorsal spinal cord surface. The Infinite Horizon (IH) impactor (Precision System and Instrumentation, LLC., Lexington, KY, USA) was used to induce compression injury with a force of 200 k dynes and a dwell time of zero seconds to the spinal cord. Care was taken to place the rats on the IH impactor table with the spinal cord parallel to it. Following the injury, the delivery of the planned force to the spinal cord was confirmed. The muscles and skin were closed in layers. The urinary bladder was manually emptied twice daily during the first week and once daily thereafter for 20 weeks. One day after the spinal cord injury procedure, two rats showing movements in their hindlimbs were excluded from the study.

### 4.3. Intravenous Administration of CL2020 (Muse Cells)

Two weeks after SCI, the Muse-cell-based product CL2020 (CL2020 group, *n* = 8) or Dulbecco’s Phosphate Buffered Saline (D-PBS, Funakoshi Co., Ltd., Tokyo, Japan) (Control group, *n* = 12) was intravenously administered through the tail vein. A total of 0.3 mL of CL2020 containing 300,000 Muse cells or D-PBS was slowly injected over 1 min. All rats received a subcutaneous injection of an immunosuppressant (Prograf, 0.5 mg/kg) (Tacrolimus Hydrate, Astellas Pharma, Inc., Tokyo, Japan) every other day for 20 weeks.

### 4.4. Behavioral Analysis

The hindlimb motor functions of all animals (*n* = 20) were evaluated using the Basso, Beattie, and Bresnahan (BBB) locomotor scale before and following SCI on days 1, 5, and 7 and weekly thereafter for 20 weeks [28]. The BBB scores were recorded by an animal care technician who was blinded to the study and animal groups. The BBB scores were compared between the CL2020 and Control groups using a multiple measurement analysis of variance (ANOVA) followed by the Bonferroni post hoc test. All values are shown as means ± standard deviations.

### 4.5. Immunohistochemical Analyses

Twenty weeks after SCI, all rats, except the animals undergoing the loss-of-function study, were anesthetized through aspiration with excess isoflurane. Rats were transcardially perfused with saline to remove blood and then with 2% paraformaldehyde (0.1 mol/L). Fixed spinal cords were cut into 10 mm pieces with the injured portion in the center and embedded to be frozen in liquid nitrogen (Thermo Scientific^TM^ Shandon^TM^ M-1 Embedding Matrix, Thermo Fisher Scientific, Waltham, MA, USA). Using a cryostat, the spinal cords were sectioned to a thickness of 5 μm. Slides were created from spinal cord sections 1, 1.5, 2, 3, 4, and 5 mm in the rostral and caudal directions, with the injured epicenter as 0, in 5 and 7 animals in the CL2020 and Control groups, respectively.

### 4.6. Cystic Cavity and Spared Spinal Cord Tissue Measurement

Hematoxylin and eosin (HE) staining was used for morphological evaluations. In the spinal cord, areas of the cystic cavity and spared spinal cord tissue were measured using ImageJ (Rasband, W.S., ImageJ, U. S. National Institutes of Health, Bethesda, MD, USA). Cystic cavity volumes were calculated using the cavity areas on slides 1, 1.5, 2, 3, 4, and 5 mm in the rostral and caudal directions, with the injured epicenter as 0. Cavity volumes were calculated in 5 and 7 animals in the CL2020 and Control groups, respectively. The results obtained were compared using the Mann–Whitney U test. Areas of spared spinal cord tissue were also calculated on slides 1, 2, 3, 4, and 5 mm in the rostral and caudal directions, with the injured epicenter as 0. Areas in the CL2020 and Control groups were compared using the Mann–Whitney U test.

### 4.7. Quantification of Preserved 5-HT Fibers

Fluorescent staining of an anti-5-HT antibody (ab66047, Abcam, Cambridge, UK) was used to evaluate preserved 5-HT fibers in the spinal cord. The number of 5-HT-immunolabeled axons 3 mm caudal to the epicenter of the injury was quantified with ImageJ (Rasband, W.S., ImageJ, U. S. National Institutes of Health, Bethesda, MD, USA) in a section perpendicular to the rostral–caudal axis of the spinal cord. The number of positive fibers was counted in three different high-magnification images from every fifth section. The number of positive fibers was calculated in a given area of 1 square millimeter during microscopic analysis. Preserved 5-HT fibers in the 5 animals in CL2020 and 7 animals in Control groups were compared using the Mann–Whitney U test.

### 4.8. Identification of Muse Cells in the Injured Spinal Cord

Immunostaining with antibodies against human mitochondria (hMit) (1:50; ab3298, Abcam, Cambridge, UK) was used to identify human Muse cells in CL2020 homing to the injured spinal cord, conjugated with Alexa 488 anti-mouse (Invitrogen, Waltham, MA, USA) as the secondary antibody. 4’,6-Diamidino-2-phenylindole (DAPI) was used to detect nuclei. Microtubule-associated protein-2 (MAP-2) (1:500; ab5392, Abcam) with a secondary antibody (Alexa 568 anti-chicken, Invitrogen), NeuN (1:100; ab177487, Abcam) with a secondary antibody (Alexa 568 anti-rabbit, Invitrogen), glial fibrillary acidic protein (GFAP) (1:200; 80788, cell signaling technology) with a secondary antibody (Alexa 568 anti-rabbit, Invitrogen), and Glutathione S-Transferase (GST)-pi (1:200; 311, Medical & Biological Laboratories) with a secondary antibody (Alexa 568 anti-rabbit, Invitrogen) were used to identify neurons, astrocytes, and oligodendrocytes, respectively. To evaluate synaptic activities, synaptophysin (1:200; MA5-14532, Invitrogen) with a secondary antibody (Alexa 568 anti-rabbit, Invitrogen) was used. Samples were enclosed with a mounting agent (Prolong diamond antifade mountant with DAPI: P36962, Thermo Fisher Scientific, Waltham, MA, USA) and inspected under a laser confocal microscope (FV3000, OLYMPUS, Tokyo, Japan).

### 4.9. Administration of Diphtheria Toxin

Twenty weeks after SCI, a loss-of-function study was performed using diphtheria toxin from Corynebacterium diphtheriae (Sigma-Aldrich Co., LLC., St. Louis, MI, USA). A previous study reported that human cells were 100,000-fold more sensitive to the toxin than rodent cells [29]. Therefore, diphtheria toxin selectively ablates human cells in rodent models [22]. Three and five rats from the CL2020 and Control groups were intraperitoneally administered diphtheria toxin (50 μg/kg) twice at a 24 h interval. Hindlimb motor functions were assessed 5 days after the administration of diphtheria toxin. A paired *t*-test was used to evaluate the BBB scores before and after the administration of diphtheria toxin.

## 5. Conclusions

Spinally injured animals achieved significant functional recovery following the intravenous administration of the human-derived product CL2020 2 weeks after the injury. In this subacute SCI model, engrafted Muse cells in CL2020 migrated and homed to the spinal cord. They differentiated into neuronal and neural cells and contributed to the restoration of descending spinal tracts. CL2020 offers a feasible treatment option in future clinical trials on SCI patients.

## Figures and Tables

**Table 1:** Proportions of the different cell types differentiated from the transplanted CL2020, found in the injured spinal cord 20 weeks after the spinal cord injury.

	MAP-2	GFAP	GST-pi
**Rostral to the injury center**	54.0 ± 3.5%	24.0 ± 2.5%	22.0 ± 1.9%
**Injury center**	48.4 ± 2.8%	26.6 ± 6.0%	25.0 ± 5.0%
**Caudal to the injury center**	52.3 ± 4.6%	25.6 ± 1.7%	22.1 ± 3.1%

n = 5, MAP-2; microtubule-associated protein-2, GFAP; glial fibrillary acidic protein, GST-pi; Glutathione S-transferase-pi.

## Data Availability

The authors confirm that the data supporting the findings of this study are available within the article.
